# Coproduction of 5-Aminovalerate and δ-Valerolactam for the Synthesis of Nylon 5 From L-Lysine in *Escherichia coli*

**DOI:** 10.3389/fbioe.2021.726126

**Published:** 2021-09-16

**Authors:** Jie Cheng, Wenying Tu, Zhou Luo, Li Liang, Xinghua Gou, Xinhui Wang, Chao Liu, Guoqiang Zhang

**Affiliations:** ^1^ Key Laboratory of Medicinal and Edible Plants Resources Development of Sichuan Education Department, Sichuan Industrial Institute of Antibiotics, Chengdu University, Chengdu, China; ^2^ Key Laboratory of Industrial Biotechnology, Ministry of Education, Jiangnan University, Wuxi, China; ^3^ National Engineering Laboratory for Cereal Fermentation Technology, Jiangnan University, Wuxi, China

**Keywords:** 5-aminovalerate, δ-valerolactam, L-lysine HCl, equilibrium mixture, H_2_O_2_

## Abstract

The compounds 5-aminovalerate and δ-valerolactam are important building blocks that can be used to synthesize bioplastics. The production of 5-aminovalerate and δ-valerolactam in microorganisms provides an ideal source that reduces the cost. To achieve efficient biobased coproduction of 5-aminovalerate and δ-valerolactam in *Escherichia coli*, a single biotransformation step from L-lysine was constructed. First, an equilibrium mixture was formed by L-lysine α-oxidase RaiP from *Scomber japonicus*. In addition, by adjusting the pH and H_2_O_2_ concentration, the titers of 5-aminovalerate and δ-valerolactam reached 10.24 and 1.82 g/L from 40 g/L L-lysine HCl at pH 5.0 and 10 mM H_2_O_2_, respectively. With the optimized pH value, the δ-valerolactam titer was improved to 6.88 g/L at pH 9.0 with a molar yield of 0.35 mol/mol lysine. The ratio of 5AVA and δ-valerolactam was obviously affected by pH value. The ratio of 5AVA and δ-valerolactam could be obtained in the range of 5.63:1–0.58:1 at pH 5.0–9.0 from the equilibrium mixture. As a result, the simultaneous synthesis of 5-aminovalerate and δ-valerolactam from L-lysine in *Escherichia coli* is highly promising. To our knowledge, this result constitutes the highest δ-valerolactam titer reported by biological methods. In summary, a commercially implied bioprocess developed for the coproduction of 5-aminovalerate and δ-valerolactam using engineered *Escherichia coli*.

## Introduction

Over the years, mounting global environmental, climate change, economic concerns, and fossil fuel sources are leading to a shift in the production of traditional bulk chemicals toward more green, renewable, economic, and sustainable routes (Wang A. et al., 2020; Gordillo Sierra and Alper, 2020; Wendisch, 2020). In many cases, the need has been partially met by biorefineries, in which microbial cell factories convert renewable feedstock resources into high-value and useful chemicals (Gao et al., 2020; Klenk et al., 2020; Youn et al., 2020; Zhang et al., 2021). While many chemicals are being developed via biotechnology, polyamide monomers are an important class of compounds (Li et al., 2020; Prell et al., 2020; Osire et al., 2021). 5-Aminovalerate (5AVA) and δ-valerolactam are attractive monomers for the production of biopolyamides, serving as raw materials for clothes, architecture, and disposable goods.

Plastics are mainly derived from petroleum feedstock. Bioplastics have attracted enormous interest because of their main degradability (Ben Abdallah et al., 2020). The annual output of bioplastics is predicted to exceed 2.43 million tons in 2024 (Haupka et al., 2020). Among microbial bioplastics, biopolyamides are widely applied in chemical, automotive, and textile industries (Ligon et al., 2017). The monomers of polyamides are primarily dicarboxylic acids, diamines, lactams, and ω-amino acids (Radzik et al., 2019). Examples of these main platform chemicals range from succinate (Zhang et al., 2009), glutarate (Zhao et al., 2018), to adipate (Wang F. et al., 2020) for dicarboxylic acids; from putrescine, cadaverine (Rui et al., 2020; Xue et al., 2020), to 1,6-hexanediamine for diamines; from δ-valerolactam (Zhang et al., 2017a), to ε-caprolactam (Thompson et al., 2020) for lactams; from 3-hydroxybutyrate (Atakav et al., 2021; Mierziak et al., 2021; Schmid et al., 2021), 2-hydroxybutyrate (Tian et al., 2021), to 3-hydroxyhexanoate (Harada et al., 2021) for hydroxyl acids; and from 4-aminobutyrate, 5AVA (Cheng et al., 2021b), to 6-aminocaproate (Turk et al., 2016) for ω-amino acids. In this respect, also 5AVA (Adkins et al., 2013) and δ-valerolactam (Xu et al., 2020) are attractive C5 platform chemicals for the production of biopolyamides from renewable biomass.

Four metabolic routes of 5AVA from L-lysine have been developed so far. The first route is the 5-aminovaleramide–mediated pathway that involves L-lysine 2-monooxygenase (DavB) and δ-aminovaleramidase (DavA) (Joo et al., 2017). The engineering WL3110 strain with overexpression of DavA and DavB generated 3.6 g/L 5AVA (Park et al., 2013). Shin et al. reported that 33.1 g/L of 5AVA was successfully formed by promoter optimization (Shin et al., 2016). The second route is the cadaverine-mediated pathway that does not require oxygen involves L-lysine decarboxylase (LdcC), putrescine transaminase (PatA), and γ-aminobutyraldehyde dehydrogenase (PatD) (Haupka et al., 2020). Haupka et al. reported that 3.7 g/L 5AVA was reached, with a yield of 0.09 g/g in shake flasks (Haupka et al., 2020). The third route is 2-keto-6-aminocaproate (2K6AC)–mediated pathway that involves L-lysine α-oxidase (RaiP) from *Scomber japonicus* (*S. japonicus*) and H_2_O_2_ (Pukin et al., 2010). Pukin et al. found that 13.4 g/L 5AVA was enzymatically produced by RaiP from *Trichoderma viride* (Pukin et al., 2010). Interestingly, Cheng et al. proposed that the titer of 5AVA could be improved to 29.12 g/L by adding 4% (v/v) ethanol and 10 mM H_2_O_2_ (Cheng et al., 2018b). Independently, a three-step route based on RaiP, α-ketoacid decarboxylase (KivD) from *Lactococcus lactis*, and aldehyde dehydrogenase (PadA) from *Escherichia coli* (*E. coli*) was established in *E. coli* with 5AVA titer up to about 52.24 g/L (Cheng et al., 2021b).

Lactams are important chemicals used in the manufacture of commercial polyamides. However, there are few reports on the direct bioproduction of lactams from engineered microorganisms. Zhang et al. confirmed that 1.1 g/L γ-butyrolactam was formed from L-glutamate by identifying a newly 2-pyrrolidone synthase ORF26 from *Streptomyces aizunensis*, with a yield of 0.14 g/g (Zhang et al., 2016). Then, Zhang et al. further revealed the catalytic promiscuity of ORF26, which cyclized ω-amino acids to produce of γ-butyrolactam, δ-valerolactam, and ε-caprolactam (Zhang et al., 2017b). However, the titers of δ-valerolactam and ε-caprolactam achieved were relatively low; 705 mg/L δ-valerolactam and 2.02 mg/L ε-caprolactam were produced, respectively. Chae et al. reported that β-alanine CoA transferase could activate ω-amino acids to produce 54.14 g/L γ-butyrolactam, 29 mg/L δ-valerolactam, and 79.6 μg/L ε-caprolactam, respectively (Chae et al., 2017). In addition, a novel route for δ-valerolactam was discovered through the direct oxidative decarboxylation of L-pipecolic acid by DavB in Xu et al.’s research (Xu et al., 2020). 90.3 mg/L δ-valerolactam was achieved from L-pipecolic acid by DavB expressed in *E. coli* (Xu et al., 2020). However, the titer of δ-valerolactam generated was rather low (Table 1).

**TABLE 1:** Production of 5AVA and δ-valerolactam in microbes.

Host strain	Strategy	5AVA titer (g/L)	Yield (g/g)	δ-Valerolactam (g/L)	Yield (g/g)	Substrate/feedstock	References
*E. coli*	Enzymatic catalysis	63.20	0.62	—	—	L-lysine	Li et al. (2016)
*C. glutamicum*	Fermentation	0.26	0.007	—	—	Rice straw hydrolysate	Sasikumar et al. (2021)
*C. glutamicum*	Fermentation	5.10	0.13	—	—	Glucose and alternative carbon sources	Jorge et al. (2017)
*C. glutamicum*	Fermentation	3.70	0.09	—	—	Glucose	Haupka et al. (2020)
*C. glutamicum*	Fed-batch fermentation	33.10	0.10	—	—	Glucose	Shin et al. (2016)
*C. glutamicum*	Fed-batch fermentation	12.51	0.10	—	—	*Miscanthus* hydrolysate	Joo et al. (2017)
*E. coli*	Whole-cell biotransformation	240.70	0.70	—	—	L-lysine	Wang et al. (2016)
*E. coli*	Whole-cell biotransformation	29.12	0.44	—	—	L-lysine HCl	Cheng et al. (2018b)
*E. coli*	Whole-cell biotransformation	52.24	0.38	—	—	L-lysine HCl	Cheng et al. (2021b)
*E. coli*	Whole-cell biotransformation	—	—	0.24	0.06	L-lysine	Xu et al. (2020)
*E. coli*	Whole-cell biotransformation	10.24	0.26	6.88	0.17	L-lysine HCl	This study

Escapin from *Aplysia californica* (*A. californica*) is an L-amino acid oxidase, which could oxidize L-lysine to produce an antimicrobial equilibrium mixture (Kamio et al., 2009). This equilibrium mixture contains cyclic form Δ^1^-piperidine-2-carboxylase (P2C), 2-hydroxy-piperidine-2-carboxylase (2HP2C) and Δ^2^-piperidine-2-carboxylase (^2^P2C) and linear form 2K6AC, 6-amino-2-hydroxy-hex-2-enolate (6A2HH2E), and 6-amino-2,2-dihydroxy-caproate (6A2DHC) (Ko et al., 2008). P2C was proved to be the dominant component of this enzymatic product at any pH using mass spectroscopy and NMR. Interestingly, this equilibrium shifts to produce relatively more ^2^P2C at more alkaline conditions, 2K6AC, 6A2HH2E, and 6A2DHC under more acidic conditions (Ko et al., 2008). The equilibrium mixture could react with H_2_O_2_ to produce 5AVA and δ-valerolactam, and its ratios are affected by pH (Kamio et al., 2009). However, the titers of 5AVA and δ-valerolactam and its ratios were not mentioned in their studies.

**FIGURE 1:**
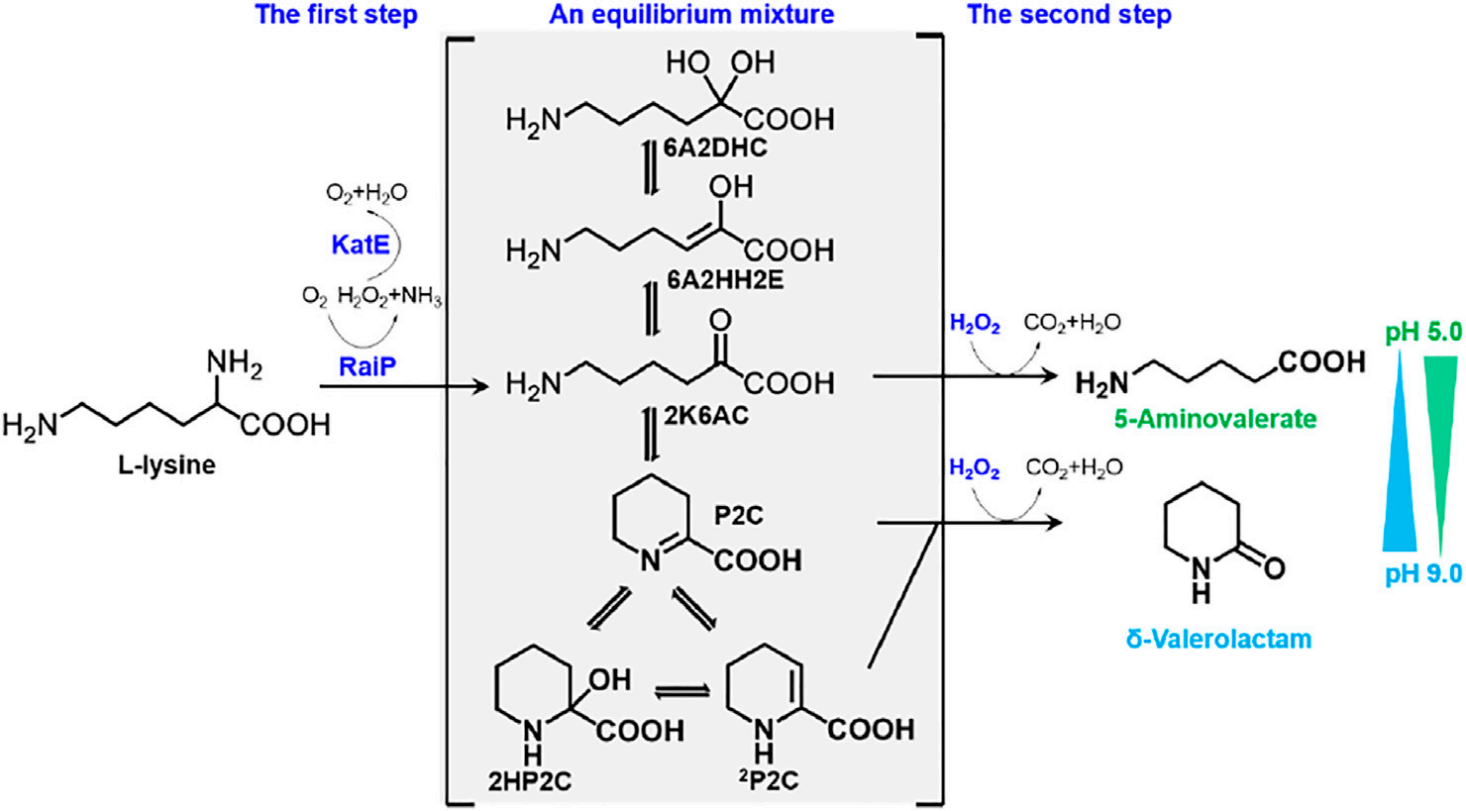
Schematic diagram of coproduction routes of 5AVA and δ-valerolactam from L-lysine in this study. RaiP, L-lysine α-oxidase; 5AVA, 5-aminovalerate; P2C, Δ^1^-piperidine-2-carboxylase; 2HP2C, 2-hydroxy-piperidine-2-carboxylase; ^2^P2C, Δ^2^-piperidine-2-carboxylase; 2K6AC, linear form 2-keto-6-aminocaproate; 6A2HH2E, 6-amino-2-hydroxy-hex-2-enolate; 6A2DHC, 6-amino-2,2-dihydroxy-caproate.

In this study, 5AVA and δ-valerolactam were coproduced from an equilibrium mixture by adjusting pH and H_2_O_2_ in *E. coli* (Figure 1). The α-amino group of L-lysine was oxidized by RaiP from *S. japonicus* to form the equilibrium mixture. 2K6AC, P2C, and ^2^P2C in this equilibrium mixture were oxidized to generate 5AVA, δ-valerolactam, and δ-valerolactam, respectively. In addition, the ratio of 5AVA and δ-valerolactam could be regulated by pH. The route of coproduction of 5AVA and δ-valerolactam was first proposed in this study. As a result, a promising strategy for coproducing 5AVA and δ-valerolactam in a single biotransformation step by adjusting the pH and H_2_O_2_ was established.

## Materials and Methods

### Strains and Plasmids

The strains and plasmids used in this work are listed in Table 2. The *raiP* from *S. japonicus* (Accession No. MG423617) was inserted into pET21a to generate plasmid pET21a-*raiP* with *Nde*I and *Bam*HI restriction sites (Cheng et al., 2018b). The gene *katE* from *E. coli* MG1655 (Accession No. AAT48137.1) was inserted into pET21a-*raiP* to generate plasmid pET21a-*raiP*-*katE* with *Sal*I and *Xho*I restriction sites. The engineered *E. coli* ML03 for knocking out lysine decarboxylase gene *cadA* was from our previous study (Cheng et al., 2018a). In addition, the plasmid pET21a, pET21a-*raiP*, and pET21a-*raiP*-*katE* were transformed into *E. coli* BL21 (DE3) or *E. coli* ML03 to obtain the strains BL21-pET21a, BL21-*raiP*, BL21-*raiP*-*katE*, ML03-pET21a, ML03-*raiP*, and ML03-*raiP*-*katE*, respectively.

**TABLE 2:** Strains and plasmids used in this study.

Strain or plasmid	Description	Sources
Strains		
DH5α	Wild type	Novagen
BL21 (DE3)	Wild type	Novagen
ML03	*E. coli* BL21 (DE3) *△cadA*	Cheng et al. (2018a)
BL21-pET21a	*E. coli* BL21 (DE3) harboring plasmid pET21a	Cheng et al. (2018b)
BL21-*raiP*	*E. coli* BL21 (DE3) harboring plasmid pET21a-*raiP*	Cheng et al. (2018b)
BL21-*raiP*-*katE*	*E. coli* BL21 (DE3) harboring plasmid pET21a-*raiP*-*katE*	This study
ML03-pET21a	*E. coli* ML03 harboring plasmid pET21a	This study
ML03-*raiP*	*E. coli* ML03 harboring plasmid pET21a-*raiP*	Cheng et al. (2018b)
ML03-*raiP*-*katE*	*E. coli* ML03 harboring plasmid pET21a-*raiP*-*katE*	This study
Plasmids		
pET21a-*raiP*	pET21a carries an L-lysine α-oxidase gene (*raiP*) from *S. japonicus*, Amp^R^	Cheng et al. (2018b)
pET21a-*raiP*-*katE*	pET21a carries an L-lysine α-oxidase gene (*raiP*) from *S. japonicus* and a catalase gene (*katE*) from *E. coli*, Kan^R^	This study

### Cultivation Conditions

The engineering strains were streaked onto Luria–Bertani (LB) agar plates with 100 mg/L Amp at 37°C for overnight. Engineering strains used for biotransformation in the shake flask were cultured in LB medium with 100 mg/L Amp. After the OD_600_ reached 0.6, 0.2 mM of isopropyl β-D-thiogalactoside (IPTG) and 6.5 g/L of L-lysine HCl were added. The pH was controlled at 5.0, 6.0, 7.0, 8.0, and 9.0 by NH_3_·H_2_O and 10% H_2_SO_4_ at 30°C after 12 h. H_2_O_2_ was added after 12 h.

### Enzyme Assays

RaiP activity was determined as Cheng et al. reported (Cheng et al., 2018b). Briefly, the reaction buffer contained 30 mM L-lysine, 26.5 mM phenol, 0.5 mM 4-aminoantipyrine, and 10 units/ml catalase. Quinoneimine dye formed was measured at 505 nm using SpectraMax M2e. One unit of enzyme activity was defined as the amount of enzyme that catalyzes the formation of 1 μM of H_2_O_2_ per minute (Cheng et al., 2018b). The activity of KatE was determined according to Liu et al. (2017); 0.1 ml diluted crude enzyme was incubated with 1 ml 60 mM H_2_O_2_ at 30°C for 10 min. The absorbance of a yellow complex formed by molybdate and H_2_O_2_ was immediately measured at 405 nm (Liu et al., 2017). One unit of catalase activity was defined as the amount of enzyme decomposing of 1 μmol H_2_O_2_ per min.

### Biotransformation

Biotransformation was performed in a 5.0-L fermenter. The medium consisted of 55 g/L of glucose, 0.004 g/L of CoCl_2_·6H_2_O, 0.02 g/L of Na_2_SO_4_, 1.6 g/L of MgSO_4_·7H_2_O, 0.0064 g/L of ZnSO_4_, 0.0006 g/L of Cu_2_SO_4_·5H_2_O, 1.6 g/L of (NH_4_)_2_SO_4_, 0.00756 g/L of FeSO_4_·7H_2_O, 2 g/L of citric acid, 7.5 g/L of K_2_HPO_4_·3H_2_O, and 250 μl of antifoam 289. The pH was controlled at 7.0 by the automatic addition of NH_3_·H_2_O and 10% H_2_SO_4_ at 30°C. After the OD_600_ reached 20, 0.2 mM IPTG was added to the broth. When the OD_600_ reached 80, the pH was controlled at 5.0, 6.0, 7.0, 8.0, and 9.0 by the automatic addition of NH_3_·H_2_O and 10% H_2_SO_4_. L-lysine HCl was added to at an initial concentration of 40 g/L. H_2_O_2_ was added after 24 h.

### Lysine, 5-Aminovalerate, and δ-Valerolactam Analysis by High-Performance Liquid Chromatography

Lysine, 5AVA, and δ-valerolactam were monitored and quantitated by high-performance liquid chromatography (HPLC). For monitoring L-lysine and 5AVA, samples were derived with phenyl isothiocyanate (PITC) with an Agilent Eclipse XDB-C18 column (4.6 mm × 150 mm × 5 μm), as described by Cheng et al. (2018b). To monitor δ-valerolactam, a Chirex®3126 (D)-penicillamine LC column (4.6 × 250 mm, Phenomenex, USA) was used (Xu et al., 2020).

## Results and Discussion

### Construction of a Synthetic Route for the Simultaneous Synthesis of 5-Aminovalerate and δ-Valerolactam in *E. coli*

**FIGURE 2:**
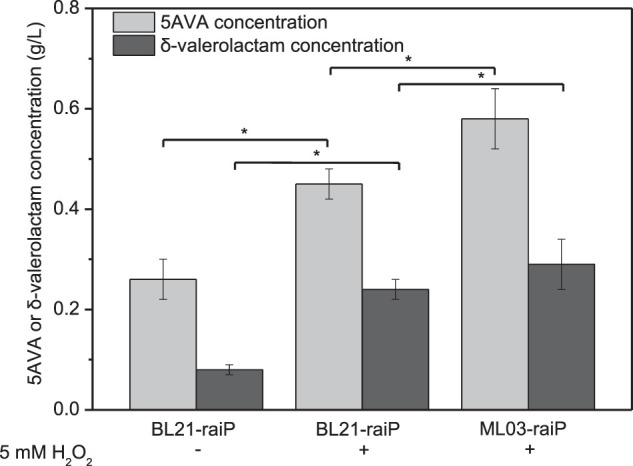
Feasibility for the coproduction of 5AVA and δ-valerolactam in a single biotransformation step. pH was controlled at 7.0. 6.5 g/L of L-lysine HCl was added as the substrate. Statistics were performed by two-tailed Student’s *t*-test. *p < 0.05. All experiments were performed a minimum of three independent sets.

A synthetic route for the concurrent synthesis of 5AVA and δ-valerolactam from L-lysine was constructed from an equilibrium mixture (Figure 1). The designed route for the coproduction of 5AVA and δ-valerolactam consists of two steps: 1) the deamination of α-amino group in L-lysine to generate an equilibrium mixture by RaiP from *S. japonicus*, with this equilibrium mixture containing P2C, 2HP2C, ^2^P2C, 2K6AC, 6A2HH2E, and 6A2DHC; 2) the decarboxylation of 2K6AC, P2C, and ^2^P2C in this equilibrium mixture to produce 5AVA and δ-valerolactam via H_2_O_2_, respectively. First, a plasmid pET21a–*raiP* was constructed and introduced into *E. coli* BL21(DE3) to obtain the strain BL21–*raiP*. As shown in Figure 2, engineering strain BL21-*raiP* produced 0.26 g/L 5AVA and 0.08 g/L δ-valerolactam in the absence of H_2_O_2,_ and 0.45 g/L 5AVA and 0.24 g/L δ-valerolactam in pH 7.0 and 5 mM H_2_O_2_, respectively. The specific activity of RaiP was 5.14 units/mg. These results demonstrated the feasibility of the coproduction of 5AVA and δ-valerolactam in *E. coli*. In addition, the strain ML03-raiP with cadA knocked out was constructed. The engineered strain ML03-*raiP* produced 0.58 g/L 5AVA and 0.29 g/L δ-valerolactam, nearly about 0.29-fold and 0.21-fold increase compared to control strain BL21-raiP at pH 7.0 and 5 mM H_2_O_2_ (Figure 2).

In the past, some studies of the concurrent bioproduction of bulk chemicals were investigated (Li et al., 2017). Few examples are the simultaneous synthesis of 5AVA and glutarate by *Corynebacterium glutamicum* (Rohles et al., 2016; Haupka et al., 2020), β-glucan and pullulan by engineering *Aureobasidium pullulans* (Wang G.-L. et al., 2020), acetoin and succinic acid by *Enterobacter cloacae* (Su et al., 2021), polyhydroxyalkanoates and exopolysaccharides by *Yangia* sp. ND199 (Romero Soto et al., 2021), and xylitol and ethanol by yeast strains (Shankar et al., 2020). Lopez-Hidalgo et al. reported that the engineered strain increased 30% the coproduction of ethanol and hydrogen used wheat straw and corn stover as feedstock (Lopez-Hidalgo et al., 2021). And 11.0 g/L polyhydroxybutyrate and 1.5 g/L violacein pigment were successfully co-synthesized in *Iodobacter* sp. PCH194 (Kumar et al., 2021). 7,12-dioxolithocholate and L-*tert*-leucine were simultaneously produced in a cofactor self-sufficient cascade system for enhancing the atom efficiency (You et al., 2021). Chae et al. found that only 29 mg/L δ-valerolactam was produced by β-alanine CoA transferase (Chae et al., 2017). Xu et al. reported that 90.3 mg/L δ-valerolactam was successfully obtained by an oxidative decarboxylase DavB (Xu et al., 2020). However, the low titers limit the prospect of industrial application.

### The Effect of H_2_O_2_ on the Simultaneous Synthesis of 5-Aminovalerate and δ-Valerolactam

**FIGURE 3:**
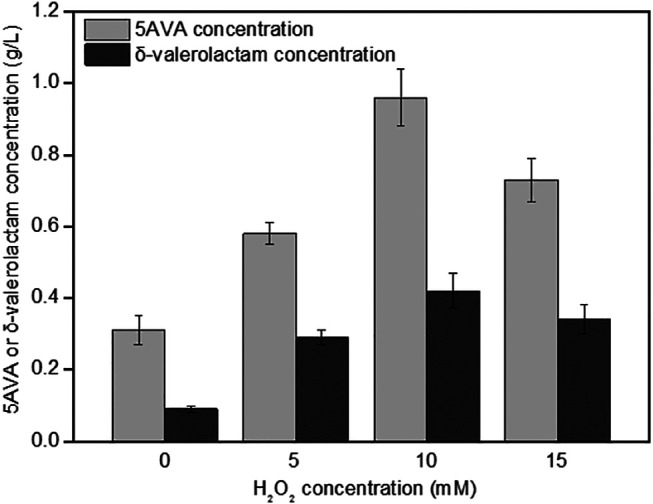
Effect of H_2_O_2_ on the coproduction of 5AVA and δ-valerolactam by strain ML03-*raiP*-*katE* in flasks. pH was controlled at 7.0. 6.5 g/L of L-lysine HCl was added as the substrate. All experiments were performed a minimum of three independent sets.

The effect of H_2_O_2_ on the simultaneous synthesis of 5AVA and δ-valerolactam in engineering strain ML03-*raiP-katE* at pH 7.0 is shown in Figure 3. It showed that the addition of H_2_O_2_ had a significant effect on the titers of 5AVA and δ-valerolactam. Engineering *E. coli* ML03-*raiP-katE* was cultured in LB medium to form an equilibrium mixture containing P2C, 2HP2C, ^2^P2C, 2K6AC, 6A2HH2E, and 6A2DHC. At 5 mM H_2_O_2_ addition, recombinant ML03-*raiP-katE* produced 0.58 g/L 5AVA and 0.29 g/L δ-valerolactam after 24 h, respectively, increased about 0.87-fold and 2.22-fold compared to the control group without H_2_O_2_. With the continuous increase in H_2_O_2_ concentration to 10 mM, the titers of 5AVA and δ-valerolactam both were further increased to 0.96 g/L 5AVA and 0.42 g/L δ-valerolactam, with a yield increase of 2.13-fold and 3.67-fold compared to the control without H_2_O_2_ addition, respectively. However, with the increase in H_2_O_2_ concentration to 15 mM, the titers of 5AVA and δ-valerolactam decreased dramatically (Figure 3).

H_2_O_2_ is an important reactive oxygen species in organisms and is produced in response to signal transduction, growth, and development (Oldroyd, 2013; Sies and Jones, 2020). H_2_O_2_ enters cells to regulate signaling and cellular processes through aquaporin membrane proteins and covalently modifies cytoplasmic proteins (Sies and Jones, 2020). Wu et al. found that H_2_O_2_ sensor HPCAI is a receptor kinase (Wu et al., 2020). However, excess of H_2_O_2_ could inhibit cell growth and affect the production of target compounds, resulting in low OD_600_ (Cheng et al., 2018b). Therefore, in this study, a strategy was proposed that the H_2_O_2_ produced by RaiP was decomposed by overexpression of catalase in *E. coli* in the early stage, and then H_2_O_2_ was added in the later stage to produce 5AVA and δ-valerolactam. The specific activity of KatE was 23.58 units/mg. The H_2_O_2_ that is generated by RaiP can affect the cell growth and the titers of products (Cheng et al., 2021b). The coexpression of RaiP and KatE in *E. coli* might provide a more convenient and effective method for the production of 5AVA and δ-valerolactam. As shown in Supplementary Figure S1, the coexpressed *E. coli* BL21 (DE3) strain harboring pET21a-*raiP*-*katE* showed another distinct 84-kDa band on SDS-PAGE, which was consistent with the calculated molecular weight of catalase.

### The Effect of pH on the Ratio of 5-Aminovalerate and δ-Valerolactam

The effect of pH on the ratio of 5AVA and δ-valerolactam in engineering strain ML03-*raiP-katE* with 10 mM H_2_O_2_ addition is shown in Table 3. It showed that the pH had a great effect on the ratio of 5AVA and δ-valerolactam; 1.12 g/L 5AVA and 0.25 g/L δ-valerolactam were generated at pH 5.0 after adding H_2_O_2_ for 12 h. The maximum ratio of 5AVA and δ-valerolactam was reached 4.48:1 at pH 5.0. With the increase in pH, the titer of δ-valerolactam increased gradually, resulting in a decrease in the ratio of 5AVA and δ-valerolactam; 1.08 g/L 5AVA and 0.33 g/L δ-valerolactam were obtained at pH 6.0. When the pH value was 7.0, recombinant ML03-*raiP-katE* could produce 0.96 g/L 5AVA and 0.42 g/L δ-valerolactam after 24 h from the equilibrium mixture. In addition, the titer of δ-valerolactam increased significantly to 0.56 g/L at pH 8.0, with a titer increase of 0.33-fold compared with pH 7.0. Interestingly, the titer of δ-valerolactam was higher than 5AVA at pH 9.0, and the ratio of 5AVA and δ-valerolactam was 0.91. As a result, the flux of the equilibrium mixture would shift to 5AVA under acidic condition and to δ-valerolactam under alkaline condition. These findings are consistent with Kamio’s research (Kamio et al., 2009). However, their specific ratio has not been reported (Ko et al., 2008; Kamio et al., 2009).

**TABLE 3:** Effect of pH on the ratio of 5AVA and δ-valerolactam in ML03-*raiP*-*katE*. Data are presented as means ± STDV calculated from three replicate biotransformation experiments. Statistics were performed by the two-tailed Student’s *t*-test. *p < 0.05; ns, not significant.

pH	Time (h)	5AVA production (g/L)	Statistical analysis^a^	δ-Valerolactam (g/L)	Statistical analysis^a^	Ratio of 5AVA and δ-valerolactam
5.0	12	0.24 ± 0.02	—	0.07 ± 0.01	—	3.42:1
	24	1.12 ± 0.07	—	0.25 ± 0.03	—	4.48:1
6.0	12	0.28 ± 0.03	ns	0.09 ± 0.01	ns	3.11:1
	24	1.08 ± 0.04	ns	0.33 ± 0.03	ns	3.27:1
7.0	12	0.31 ± 0.03	ns	0.09 ± 0.01	ns	3.44:1
	24	0.96 ± 0.04	*	0.42 ± 0.03	*	2.29:1
8.0	12	0.30 ± 0.03	ns	0.08 ± 0.01	ns	3.75:1
	24	0.92 ± 0.05	ns	0.56 ± 0.04	*	1.64:1
9.0	12	0.27 ± 0.02	ns	0.06 ± 0.01	ns	4.50:1
	24	0.68 ± 0.05	*	0.75 ± 0.05	*	0.91:1

a Statistical analysis of the 5AVA production was performed with every two separated lines. 6.5 g/L L-lys HCl and 0.2 mM IPTG were added. 10 mM H_2_O_2_ was added after 12 h.

### Biotransformation for the Coproduction of 5-Aminovalerate and δ-Valerolactam

**FIGURE 4:**
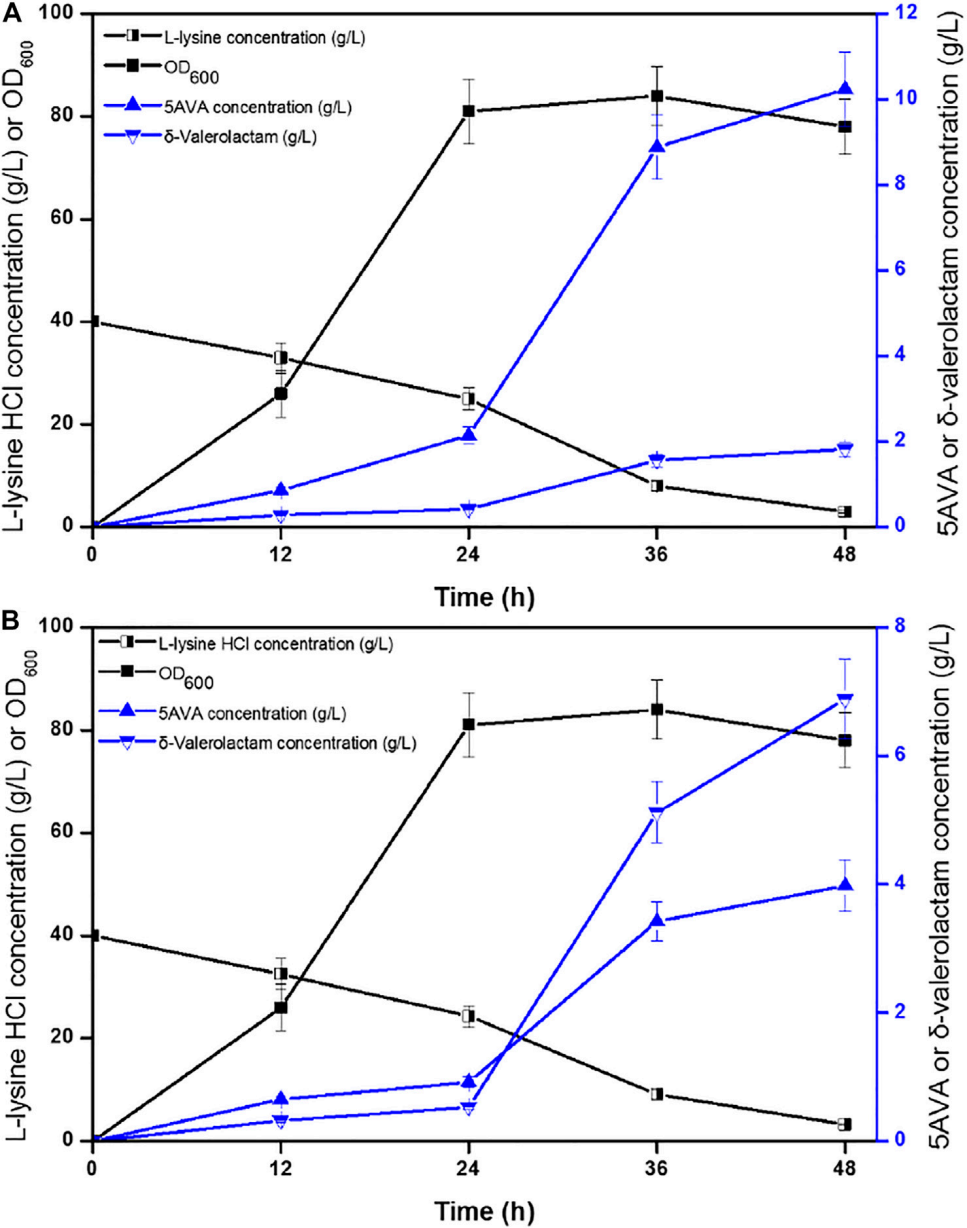
Time profiles of 5AVA and δ-valerolactam production were investigated by biotransformation of engineered strain ML03-*raiP*-*katE* at pH 5.0 **(A)** or pH 9.0 **(B)** in a 5-L fermenter. The experiments were conducted at 40 g/L L-lysine HCl, 37°C and 250 rpm. 10 mM H_2_O_2_ was added after reaction 24 h. All experiments were performed a minimum of three independent sets.

Time profiles for the simultaneous synthesis of 5AVA and δ-valerolactam were investigated by biotransformation of engineered strain ML03-*raiP*-*katE* at pH 5.0 (Figure 4A) and pH 9.0 (Figure 4B) in a 5-L fermenter. The catalase KatE was overexpressed to remove H_2_O_2_, which significantly improved OD_600_ and the titer of products in the 5-L fermenter (Cheng et al., 2021b). The titers of 5AVA and δ-valerolactam were very low before the addition of H_2_O_2_. In this process, the main accumulation was the equilibrium mixture produced by RaiP from lysine. Although H_2_O_2_ was produced by RaiP, its low concentration leads to low production of 5AVA and δ-valerolactam. After adding H_2_O_2_ for 12 h, the titers of 5AVA and δ-valerolactam increased significantly to 8.88 and 1.56 g/L at pH 5.0. Finally, 10.24 g/L 5AVA and 1.82 g/L δ-valerolactam were obtained, with a total molar yield of 0.52 mol/mol lysine, and its ratio was 5.63:1 at pH 5.0. The difference was that the titers of 5AVA and δ-valerolactam were 3.42 and 5.12 g/L after adding H_2_O_2_ for 12 h at pH 9.0. Finally, 3.98 g/L 5AVA and 6.88 g/L δ-valerolactam were obtained, with a total molar yield of 0.51 mol/mol lysine, and its ratio was 0.58:1 at pH 9.0. The previous results showed that the ratio of 5AVA and δ-valerolactam was significantly regulated by pH. δ-Valerolactam would be the main component in alkaline condition.

We have previously reported the production of 5AVA by overexpression of RaiP, but the titer and change in δ-valerolactam were not noticed in this process. At the same time, the addition of ethanol improved the expression level of RaiP, which increases the cost and leads to uneconomical (Cheng et al., 2018b; Cheng et al., 2020; Cheng et al., 2021a). Xu et al. reported that the expression of DavB from *P. putida* could synthesize 90.3 mg/L of δ-valerolactam from L-pipecolic acid (Xu et al., 2020). Interestingly, the coexpression of RaiP, glucose dehydrogenase GDH, P2C reductase DpkA, and LysP could produce more δ-valerolactam from lysine, up to 242 mg/L (Xu et al., 2020). This may be due to the fact that part of δ-valerolactam does not originate from the oxidative decarboxylation of L-pipecolic acid but from this equilibrium mixture in this study. Compared with other biotransformation for production of 5AVA, the advantage in this study was to realize the simultaneous synthesis of 5AVA and δ-valerolactam. In terms of biotransformation mechanism, the simultaneous synthesis of 5AVA and δ-valerolactam mainly includes two steps: 1) the formation of an equilibrium mixture by RaiP from lysine and 2) the oxidization of the equilibrium mixture to 5AVA and δ-valerolactam by H_2_O_2_ at different pH values.

## Conclusion

Many important monomers of polyamides, such as adipate, cadaverine, and 3-hydroxybutyrate, have been extensively studied in microbes. The results presented here demonstrated that engineering *E. coli* also has the potential to be used as a promising alternative to produce monomers of polyamides derived from petrochemicals. In this study, the strategy for coproducing 5AVA and δ-valerolactam by adjusting the pH and H_2_O_2_ in *E. coli* was proposed. H_2_O_2_ was regulated to improve the synthesis efficiency of δ-valerolactam in *E. coli* in different pH environments, which also increased 5AVA accumulation. The ratio of 5AVA and δ-valerolactam was significantly affected by pH value. δ-Valerolactam would be the main component in alkaline condition. The titers of 5-aminovalerate and δ-valerolactam reached 3.98 and 6.88 g/L from 40 g/L L-lysine HCl at pH 9.0, with a total yield of 0.51 mol/mol lysine. The present findings indicated a promising strategy for the simultaneous synthesis of two commercial products in a single biotransformation step. These strategies could be widely applied for sustainable production of many commercially monomers of polyamides.

## Data Availability

The original contributions presented in the study are included in the article/Supplementary Material; further inquiries can be directed to the corresponding authors.
