# Supplementary material for: Coproduction of 5-Aminovalerate and δ-Valerolactam for the Synthesis of Nylon 5 From L-Lysine in Escherichia coli
Source: Front Bioeng Biotechnol. 2021 Sep 16;9:726126. doi: 10.3389/fbioe.2021.726126 (PMC8481640; doi:10.3389/fbioe.2021.726126)
Supplement: Supplementary file 1 [file DataSheet1.docx]

# Supporting information for:

**Coproduction of 5-****Aminovalerate and δ-Valerolactam for the Synthesis of Nylon 5 from L-Lysine in *Escherichia coli***

**Jie Cheng^1^, Wenying Tu^1,2^,** **Zhou Luo^1,3^, Li Liang^1^, Xinghua Gou^1^, Xinhui Wang^1^, Chao Liu^2^*, Guoqiang Zhang^3^***

^1^*Key Laboratory of Medicinal and Edible Plants Resources Development of Sichuan Education Department, Sichuan Industrial Institute of Antibiotics, Chengdu University, Chengdu 610106, P. R. China*

^2^*Key Laboratory of Industrial Biotechnology, Ministry of Education, Jiangnan University, Wuxi 214122, P. R. China*

^3^*National Engineering Laboratory for Cereal Fermentation Technology, Jiangnan University, Wuxi 214122, P. R. China*

^*^ Address Correspondence:

Key Laboratory of Industrial Biotechnology, Ministry of Education, Jiangnan University, 1800 Lihu Road, Wuxi 214122, P. R. China.

National Engineering Laboratory for Cereal Fermentation Technology, Jiangnan University, 1800 Lihu Road, Wuxi 214122, P. R. China.

***E-mail*:** [histliuchao@163.com](mailto:histliuchao@163.com) (C. Liu); [gqzhang@jiangnan.edu.cn](mailto:gqzhang@jiangnan.edu.cn) (G. Zhang)

**Supplementary S1**

**Nucleotide sequence of codon optimized *raiP* from *S. japonicas* (Accession No. MG423617) used in this study is listed as follows:**

ATGGAGCACCTGGCAGACTGTCTGGAGGACAAAGACTACGACACACTGCTGCAAACCCTGGATAATGGCCTGCCGCACATCAATACCAGCCACCACGTGGTTATCGTGGGTGCAGGCATGGCAGGCCTGACAGCCGCAAAGCTGTTACAAGACGCAGGCCATACCGTGACCATTCTGGAAGCAAATGATCGCGTGGGCGGCCGCGTGGAAACCTATCGCAACGAGAAAGAGGGCTGGTACGCAGAAATGGGCGCCATGCGCATTCCGAGCAGCCACCGCATCGTTCAGTGGTTTGTGAAGAAGCTGGGCGTTGAGATGAACGAGTTTGTGATGACCGACGACAACACCTTCTATCTGGTGAATGGCGTGCGTGAGCGCACCTATGTGGTGCAGGAGAACCCTGATGTGCTGAAATACAATGTGAGCGAAAGCGAAAAAGGCATTAGTGCCGACGACCTGCTGGACCGTGCCTTACAAAAGGTGAAAGAAGAGGTGGAAGCCAATGGCTGCAAAGCAGCCCTGGAGAAGTATGACCGCTATAGCGTTAAAGAATATCTGAAAGAAGAAGGTGGTCTGAGTCCGGGTGCCGTGCGTATGATCGGCGATCTGCTGAACGAACAGAGCCTGATGTATACCGCACTGAGCGAAATGATCTATGATCAGGCAGATGTGAACGACAGCGTGAGTTATCACGAAGTGACCGGTGGTAGTGACCTGCTGCCGGAAGCCTTTCTGAGCGTGCTGGACGTGCCGATCCTGCTGAACAGCAAAGTGAAACATATCCGCCAGAGCGATAAGGGTGTGATCGTGAGCTATCAGACAGGCAACGAAAGCAGCCTGATGGATCTGAGCGCAGATATTGTGCTGGTGACCACCACCGCCAAAGCCGCCCTGTTTATCGACTTCGATCCTCCGCTGAGCATCAGCAAAATGGAGGCCCTGCGCAGCGTGCACTATGATAGCAGCACCAAGATCCTGCTGACCTTCCGCGACAAGTTCTGGGAAGATGACGGTATCCGTGGCGGCAAAAGCATTACAGACGGTCCGAGCCGCTACATTTACTACCCGAGCCACAGCTTTCACACCAACGAAACCATTGGTGTGCTGCTGGCCAGTTATACCTGGAGTGACGAAAGTCTGCTGTTCCTGGGTGCCAGCGATGAGGAGCTGAAAGAGCTGGCCCTGCGTGATCTGGCAAAGATTCATGGTGAACAGGTGTGGGACAAGTGCACCGGCGTGATCGTGAAAAAGTGGAGTGCAGACCCGTATAGCCTGGGCGCCTTTGCCCTGTTCACACCGTACCAGCATCTGGAGTATGCCCAGGAACTGTTTAGCAGTGAGGGTCGTGTGCACTTTGCCGGTGAACATACCGCCTTTCCGCATGCCTGGATCGAGACCAGCATGAAAAGTGCCATCCGCGCCGCCACCAACATTAACAAAGTGGCAAATGAAGAAAGCACCATCGAGCATACCAAGGACGAACTGTAA

**Nucleotide sequence of *katE* from *E. coli* MG1655 (Accession No. AAT48137.1) used in this study is listed as follows:**

ATGTCGCAACATAACGAAAAGAACCCACATCAGCACCAGTCACCACTACACGATTCCAGCGAAGCGAAACCGGGGATGGACTCACTGGCACCTGAGGACGGCTCTCATCGTCCAGCGGCTGAACCAACACCGCCAGGTGCACAACCTACCGCCCCAGGGAGCCTGAAAGCCCCTGATACGCGTAACGAAAAACTTAATTCTCTGGAAGACGTACGCAAAGGCAGTGAAAATTATGCGCTGACCACTAATCAGGGCGTGCGCATCGCCGACGATCAAAACTCACTGCGTGCCGGTAGCCGTGGTCCAACGCTGCTGGAAGATTTTATTCTGCGCGAGAAAATCACCCACTTTGACCATGAGCGCATTCCGGAACGTATTGTTCATGCACGCGGATCAGCCGCTCACGGTTATTTCCAGCCATATAAAAGCTTAAGCGATATTACCAAAGCGGATTTCCTCTCAGATCCGAACAAAATCACCCCAGTATTTGTACGTTTCTCTACCGTTCAGGGTGGTGCTGGCTCTGCTGATACCGTGCGTGATATCCGTGGCTTTGCCACCAAGTTCTATACCGAAGAGGGTATTTTTGACCTCGTTGGCAATAACACGCCAATCTTCTTTATCCAGGATGCGCATAAATTCCCCGATTTTGTTCATGCGGTAAAACCAGAACCGCACTGGGCAATTCCACAAGGGCAAAGTGCCCACGATACTTTCTGGGATTATGTTTCTCTGCAACCTGAAACTCTGCACAACGTGATGTGGGCGATGTCGGATCGCGGCATCCCCCGCAGTTACCGCACCATGGAAGGCTTCGGTATTCACACCTTCCGCCTGATTAATGCCGAAGGGAAGGCAACGTTTGTACGTTTCCACTGGAAACCACTGGCAGGTAAAGCCTCACTCGTTTGGGATGAAGCACAAAAACTCACCGGACGTGACCCGGACTTCCACCGCCGCGAGTTGTGGGAAGCCATTGAAGCAGGCGATTTTCCGGAATACGAACTGGGCTTCCAGTTGATTCCTGAAGAAGATGAATTCAAGTTCGACTTCGATCTTCTCGATCCAACCAAACTTATCCCGGAAGAACTGGTGCCCGTTCAGCGTGTCGGCAAAATGGTGCTCAATCGCAACCCGGATAACTTCTTTGCTGAAAACGAACAGGCGGCTTTCCATCCTGGGCATATCGTGCCGGGACTGGACTTCACCAACGATCCGCTGTTGCAGGGACGTTTGTTCTCCTATACCGATACACAAATCAGTCGTCTTGGTGGGCCGAATTTCCATGAGATTCCGATTAACCGTCCGACCTGCCCTTACCATAATTTCCAGCGTGACGGCATGCATCGCATGGGGATCGACACTAACCCGGCGAATTACGAACCGAACTCGATTAACGATAACTGGCCGCGCGAAACACCGCCGGGGCCGAAACGCGGCGGTTTTGAATCATACCAGGAGCGCGTGGAAGGCAATAAAGTTCGCGAGCGCAGCCCATCGTTTGGCGAATATTATTCCCATCCGCGTCTGTTCTGGCTAAGTCAGACGCCATTTGAGCAGCGCCATATTGTCGATGGTTTCAGTTTTGAGTTAAGCAAAGTCGTTCGTCCGTATATTCGTGAGCGCGTTGTTGACCAGCTGGCGCATATTGATCTCACTCTGGCCCAGGCGGTGGCGAAAAATCTCGGTATCGAACTGACTGACGACCAGCTGAATATCACCCCACCTCCGGACGTCAACGGTCTGAAAAAGGATCCATCCTTAAGTTTGTACGCCATTCCTGACGGTGATGTGAAAGGTCGCGTGGTAGCGATTTTACTTAATGATGAAGTGAGATCGGCAGACCTTCTGGCCATTCTCAAGGCGCTGAAGGCCAAAGGCGTTCATGCCAAACTGCTCTACTCCCGAATGGGTGAAGTGACTGCGGATGACGGTACGGTGTTGCCTATAGCCGCTACCTTTGCCGGTGCACCTTCGCTGACGGTCGATGCGGTCATTGTCCCTTGCGGCAATATCGCGGATATCGCTGACAACGGCGATGCCAACTACTACCTGATGGAAGCCTACAAACACCTTAAACCGATTGCGCTGGCGGGTGACGCGCGCAAGTTTAAAGCAACAATCAAGATCGCTGACCAGGGTGAAGAAGGGATTGTGGAAGCTGACAGCGCTGACGGTAGTTTTATGGATGAACTGCTAACGCTGATGGCAGCACACCGCGTGTGGTCACGCATTCCTAAGATTGACAAAATTCCTGCCTGA

**Supplementary Figure S1.** Expression analysis of RaiP and KatE.


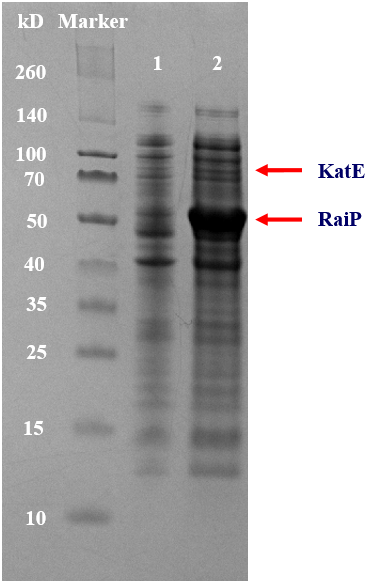


Figure S1. Expression analysis of RaiP and KatE. Protein samples were separated by 12% SDS-PAGE and stained with coomassie brilliant blue. Lane 1, noninduced control; Lane 2: The expression was induced with 0.2 mM IPTG.

The sizes of the recombinant proteins were 55 and 84 kDa respectively, which were consistent with the predicted size of RaiP and KatE proteins.

RaiP activity was determined by measuring the rate of H_2_O_2_ formation as Cheng et al. reported. (Cheng et al. 2018b) The reaction buffer (2 mL) contained 50 mM KPB (pH 8.0), 30 mM L-lysine, 26.5 mM phenol, 0.5 mM 4-aminoantipyrine and 10 units/mL catalase. The standard reaction mixture (205 μL) contained 180 μL reaction buffer and 25 μL enzyme. The reaction was carried out at 30°C for 10 min and stopped by the addition of 10 μL of 10 mM HCl. After neutralization with 10 μL of 10 mM NaOH, Quinoneimine dye formed was measured at 505 nm using SpectraMax M2e (American Molecular Devices). One unit of enzyme activity was defined as the amount of enzyme that catalyzes the formation of 1 μmol/L of H_2_O_2_ perminute. The specific activity of RaiP was 5.14 units/mg. The activity of KatE was determined according to Liu ([Liu et al., 2017](#_ENREF_23)). 0.1 mL diluted crude enzyme was incubated with 1 mL 60 mM H_2_O_2_ at 30°C for 10 min. The enzymatic reaction was then stopped by the addition of 1 ml 32.4 mM ammonium molybdate. The absorbance of a yellow complex formed by molybdate and H_2_O_2_ was immediately measured at 405 nm ([Liu et al., 2017](#_ENREF_23)). One unit of catalase activity was defined as the amount of enzyme decomposing of 1 μmol H_2_O_2_ per min.

**Supplementary table S1.** Primers used in this study

| Primers names | Nucleotide sequence (5’-3’) |
| --- | --- |
| raiP-F | 5’-GGAATTCCATATGGAGCACCTGGCAGACTGTCTGG-3’ |
| raiP-R | 5’-CGGGATCCCAGTTCGTCCTTGGTATGC-3’ |
| katE-F | 5’-ACGCGTCGACATGTCGCAACATAACGAAAAG-3’ |
| katE-R | 5’-CCGCTCGAGTCAGGCAGGAATTTTGTCAATC-3’ |
